# Pharmacological treatments for eating disorders – How far are we from new solutions?

**DOI:** 10.1192/j.eurpsy.2025.1415

**Published:** 2025-08-26

**Authors:** V. A. Voicu, O. Vasiliu

**Affiliations:** 1 Carol Davila University of Medicine and Pharmacy; 2 Romanian Academy of Medical Sciences; 3 Romanian Academy; 4 Psychiatry Dept., Dr. Carol Davila University Emergency Central Military Hospital, Bucharest, Romania

## Abstract

**Introduction:**

Eating disorders (EDs) are an important and highly prevalent class of disorders worldwide, with the global lifetime prevalence reaching more than 8% in females and 2% in males. Also, EDs associate high rates of mortality, comorbidity, organic and psychiatric complications, and years lived with disability (YLD), low quality of life and increased healthcare costs. Yet, the pharmacological armamentarium available to treat EDs is quite reduced, a phenomenon that creates difficult challenges for the case management of these patients.

**Objectives:**

To review the existing literature in order to find new targets explored for the treatment of EDs.

**Methods:**

A literature review was conducted in four electronic databases (PubMed, EMBASE, Cochrane, and Clarivate/Web of Science) and the US National Library of Medicine database for clinical trials (www.clinicaltrials.gov) to find clinical studies published between January 2000 and September 2024. The keywords used were “eating disorders”, “feeding disorders”, “anorexia nervosa”, “bulimia nervosa”, “binge eating disorder”, “drugs in pipeline”, and “pharmacological treatment”. Only adult participants were included, and only sources published in English were selected.

**Results:**

The following data are based on 33 sources found during the literature search. Lisdexampheatmine and vortioxetine are explored for in BED, with the first agent receiving FDA approval for moderate and severe binge eating disorder (BED) in adults. Olanzapine is explored in patients with anorexia nervosa (AN), chromium picolinate for BED, methylphenidate + CBT also for BED, and antibiotics for bulimia nervosa (BN). Psychedelics, e.g., psilocybin and MDMA, are explored for AN and BED, but there is a limited number of trials dedicated to these agents that reach only phase 2a. Therapeutic guidelines dedicated to EDs have very few level A recommendations, i.e., fluoxetine and topiramate for BED, lisdexamphetamine for BED, and olanzapine for AN, with several cautionary notes regarding adverse events and restricted availability for some of these drugs.

**Conclusions:**

There is a dearth of good-quality data regarding the efficacy and tolerability of newer pharmacological agents for treating EDs. Therapeutic guidelines dedicated to this type of disorders have only scarce recommendations regarding pharmacotherapy, and there is an urgent need to find more agents that could improve the prognosis of patients with ED.

**Disclosure of Interest:**

None Declared

